# Practical Notes and Formulæ

**Published:** 1882-05-20

**Authors:** 


### PRACTICAL NOTES AND FORMULÆ.

**Ergot for Lead Poisoning.**—After long experience with this disease, in which (1) iodide of potassium alone; (2) electricity with tonics and nux vomica; (3) iodide of potassium and ergot have been fully tried, the conclusion was reached that the last was by far the most efficacious, usually enabling the patients to resume work in the mines in a month, whilst they were generally disabled for three months when treated without the ergot, though when the attack is a mild one, sulphate of magnesia followed by iodide of potassium usually relieves in a few days. The combination used is this:

℞ Potassii iodidi.................................. ʒij,

Ext. ergotæ fluidi.............................. ℥j,

Ext. nucis vomicæ.............................. ʒj,

Tr. cardamoni comp............................ ℥j,

Syrupi q. s. ad.................................. ℥iv.

M. Sig. Take a tablespoonful night and morning.—*Dr. J. A. Stiles, Therapeutic Gazette, Oct., 1881.*

**To Prevent Pitting after Small-Pox.**—We clip the following from Martin's Chemists' and Druggists' Bulletin, October, 1881.

℞ Carbolic acid.............................. ʒj to ʒiiss.

Olive oil.................................. ℥ ij,

Prepared chalk............................ ʒij.

M. Apply to the face by means of a linen mask, having openings for the eyes, nose and mouth. Suppuration is less in duration and intensity than upon portions of the body left uncovered; where the stage of suppuration begins on the thirteenth to the fifteenth day, upon the face it occurs on the ninth to the eleventh day. The mask is generally removed when desication commences. Schwienmer, in *L'Union Med. du Canada.—Ame. Med. Jour.*

**Iodine in Typhoid Fever.**—Dr. Davis' formula for administering iodine in typhoid fever, is the following:

℞. Iodinii.................................. gr. viij.

Potassii iodidi............................ gr. xxx.

Aquæ distilatæ............................ f℥ iss. M.

The dose was generelly diluted with two tablespoonfuls of sweetened water, and repeated every four hours, for the first three or four days, and then every six hours until indications of convalescence appeared.—*Med. and Surg. Reporter.*

**Diarrhea in Typhoid.**—The excessive diarrhea of typhoid is said to be remarkably controlled by the administration of twenty drops of turpentine every two or three hours.—*New York Med. Record.*

**Scrotal Pruritus.**—A subscriber in California sends the following in answer to query in December number:

EDITOR MEDICAL TIMES—*Dear Sir:* In the December number of your journal, M. D. asks for a remedy for scrotal pruritus. If he will try the following, it will cure it:

| | | |
|---|---|---|
| R. | Hydrarg. chlor. col | gr. viii. |
| | Spirits lavend. c | ʒ ss. |
| | Aqua | ℥ viii. |

M. Apply three times a day. I have cured a great many cases with the above. One of the worst was a gentleman from Chicago who was troubled six years, and had tried several physicians there without permanent relief.—*Chicago Med. Times.*

**Colicky Babies.**—A writer in Chicago Medical Times suggests the following:

| | | |
|---|---|---|
| ℞. | Potassii bromidi | ʒ j. |
| | Ol. anisi | m j. |
| | Mucil. acasiæ | ℥ ij. |
| | Glycerini | ʒ ij. |
| | Aquæ | ℥ ss. M. |

Of which ateaspoonful may be given when the c olic comes on. We may order it without fear, knowing that it ıs perfectly safe and can do no mischief, which cannot be said of the various soothing combinations and carminatives in common use in the nursery which usually contain laudnum or morphia. In a former generation it was Godfrey's cordial that was popular; now it is Mrs. Winslow's soothing syrup; but the anodyne is the same in a different form.

**Grease Eradicator.**—Kilner (Boston Journal of Chemistry) gives the following recipe for this compound—

| | | |
|---|---|---|
| ℞ | Castile soap, in shavings | ℥jv, |
| | Carbonate of soda, powdered, | ℥ij, |
| | Borax, powdered, | ʒj, |
| | Aqua ammonia, | ℥vij, |
| | Alcohol, | ℥iij, |
| | Turpentine, | ℥ij, |
| | Sulphuric acid, | ℥ij. |

**Dr, Hamilton's Prescription for Epilepsy.**—

| | | |
|---|---|---|
| ℞ | Strychniæ sulph | gr. i, |
| | Fl. ext. ergotæ, | ℥ ss, |
| | Lig. potass. arsenit, | ʒ ij, |
| | Sodi bromid | ℥ ss, |
| | Tr. digitalis, | ʒ iij, |
| | Aquæ menth. pip. ad | ℥ iv. |

M. Sig. A teaspoonful before eating in a half tumblerful of water.—*Mich. Med. News.*

